# Stakeholders’ perspectives on the development of an Africa-focused postgraduate diploma to address public mental health training needs in Africa: a qualitative study

**DOI:** 10.1186/s12888-023-04751-7

**Published:** 2023-04-25

**Authors:** Claire van der Westhuizen, Marlise Richter, Ashraf Kagee, Rizwana Roomaney, Marguerite Schneider, Katherine Sorsdahl

**Affiliations:** 1 Alan J Flisher Centre for Public Mental Health, Department of Psychiatry and Mental Health, University of Cape Town, 46 Sawkins Road, Cape Town, 7700 South Africa; 2 Alan J Flisher Centre for Public Mental Health, Department of Psychology, Stellenbosch University, Stellenbosch, South Africa; 3 African Centre for Migration & Society, University of the Witwatersrand, Johannesburg, South Africa

**Keywords:** Public mental health, Capacity building, Mental health research, Africa

## Abstract

**Background:**

Despite the significant contribution of mental health conditions to the burden of disease, there is insufficient evidence from Africa to inform policy, planning and service delivery. Thus, there is a need for mental health research capacity building, led by African public mental health researchers and practitioners, to drive local research priorities. The aim of African mental health Researchers Inspired and Equipped (ARISE) was to develop a one-year postgraduate diploma (PGDip) in public mental health to address the current gaps in public mental health training.

**Methods:**

Thirty-six individual interviews were conducted online with three groups of participants: course convenors of related PGDips in South Africa, course convenors of international public mental health degree programmes and stakeholders active in public mental health in Africa. The interviewers elicited information regarding: programme delivery, training needs in African public mental health, and experiences of facilitators, barriers and solutions to successful implementation. The transcribed interviews were analysed by two coders using thematic analysis.

**Results:**

Participants found the Africa-focused PGDip programme acceptable with the potential to address public mental health research and operational capacity gaps in Africa. Participants provided several recommendations for the PGDip, including that: (i) the programme be guided by the principles of human rights, social justice, diversity and inclusivity; (ii) the content reflect African public mental health needs; (iii) PGDip faculty be skilled in teaching and developing material for online courses and (iv) the PGDip be designed as a fully online or blended learning programme in collaboration with learning designers.

**Conclusions:**

The study findings provided valuable insight into how to communicate key principles and skills suited to the rapidly developing public mental health field while keeping pace with changes in higher education. The information elicited has informed curriculum design, implementation and quality improvement strategies for the new postgraduate public mental health programme.

## Background

Mental health conditions make an increasingly significant contribution to the burden of disease in African countries, yet health systems are poorly resourced to address the problem [1]. With the predicted population growth in the African region, the relatively low expenditure on mental health and the scarcity of mental health professionals, the burden of mental and substance use conditions could have a significant negative impact on health systems and African economies, particularly with the added mental health risks associated with the COVID-19 pandemic [1–5].

Although it is imperative that policymakers and practitioners are equipped to advocate for increased resources, as well as plan and implement cost-effective, locally relevant solutions there is often insufficient local evidence to inform mental healthcare planning and service provision [6, 7]. Thus, there is a need for mental health research capacity building in Africa, led by African public mental health researchers and practitioners, to drive local research priorities and retain precious human resources on the continent [8, 9].

Limited public mental health research training programmes are available on the African continent, with notable exceptions being: (i) the Mental Health Leadership and Advocacy Programme based at the University of Ibadan, Nigeria [10], (ii) the Leadership in Mental Health, Eastern Mediterranean Region course at the American University in Cairo [11] and (iii) the Masters and PhD programmes at the Alan J Flisher Centre for Public Mental Health (CPMH), led jointly by faculty from the University of Cape Town and Stellenbosch University based in South Africa [12, 13].The Masters and PhD degrees at CPMH provide clinicians, lecturers and academics, health service managers, policy makers and community organization workers with crucial skills to conduct research on various aspects of public mental health in Africa; plan and evaluate the services that they deliver and manage; lobby effectively for mental health; and take on leadership roles in the strengthening of mental health systems. Since 2012, 43 Masters and 9 doctoral students have completed their postgraduate degrees at CPMH, a number of whom subsequently attained leadership roles and remain active in public mental health research (personal communication, Sorsdahl).

Further, as mental health has gained traction in Africa as a priority health condition, CPMH has witnessed an increase in the demand for public mental health research training with a large number of PhD and Masters enquiries from sub-Saharan African countries and elsewhere. However, a number of applicants and individuals enquiring about Masters and PhD degrees do not yet have the appropriate postgraduate training to qualify for the programme, while others have clinical postgraduate training only. Thus, these applicants often lack fundamental research skills and the understanding needed to critically evaluate and apply research findings, as well as conduct basic public mental health research. Additionally, over the nine years of offering the Masters and PhD degrees in public mental health research, the faculty have found that many of these candidates require high levels of support and additional time to complete the degree, thus demonstrating the need for a foundational postgraduate diploma (PGDip) programme in public mental health research. Such a programme would provide basic public mental health and research training for policymakers, practitioners and aspiring researchers. A PGDip would also act as a pipeline to the Masters degree and facilitate improved throughput for the Masters programme.

The overall aim of the African mental health Researchers Inspired and Equipped (ARISE) project was to develop a one-year PGDip in public mental health to address the gaps in public mental health training in Africa. ARISE builds on African-led collaborative research initiatives focussed on strengthening global mental health research capacity, including the Collaborative Hubs for International Research on Mental Health (Schneider et al., 2016) and the African Mental Health Research Initiative (AMARI; Chibanda et al., 2020). The objectives of the present study were formulated to further delineate public mental health capacity building needs and guide the development of a programme to address these needs. The objectives are: (i) to explore the structure, content and delivery of current PGDip programmes offered by South African universities in health-related fields, and public mental health postgraduate programmes offered by universities in other countries; and (ii) to identify and address potential barriers and facilitators to the successful development and implementation of a pipeline PGDip programme in public mental health in Africa.

## Methods

### Study design, sampling and procedure

We used qualitative descriptive methods to address the objectives. The study is reported according to the consolidated criteria for reporting qualitative research (COREQ) [14]. Non-probability sampling, namely convenience and snowball sampling were used to recruit participants. We developed a sampling frame by conducting a desktop review of existing PGDips in related disciplines in South Africa as well as existing postgraduate programmes in public mental health or global mental health internationally, to identify course convenors as potential research participants for the qualitative interviews. Researchers also engaged the Centre’s established African networks and invited relevant stakeholders to participate in the research project. Additionally, during the interviews participants were asked to recommend other potential participants suited to the research. In total, 56 course convenors and key stakeholders were invited for an interview via an email in which the purpose of the study was explained.

### Participants

In total, we conducted 36 individual interviews of up to an hour via a video conferencing platform with: (i) course convenors in South Africa who administered PGDips in related disciplines (such as public health and medical anthropology), (ii) course convenors from universities in the United Kingdom that presented postgraduate programmes in public mental health or global mental health, and (iii) stakeholders active in public mental health in Africa. Overall, 12 male and 24 female participants from mental health (n = 17), health (n = 14) and social science (n = 5) disciplines completed an interview. Of the participants, 18 were course convenors of South African PGDip programmes, five convened international postgraduate programmes and 13 were stakeholders active in the African public mental health field from 7 African LMICs and 2 high-income countries from other regions, namely North America and Europe.

### Interviews

The two female researchers who conducted the interviews hold PhD degrees and have experience teaching postgraduate students in public health and public mental health, as well as in supervising postgraduate public mental health research. At the time of the interviews both researchers were employed within CPMH and were involved in designing and conducting formative work for the PGDip programme. One of the researchers is the course convenor of the Master of Philosophy (MPhil) in Public Mental Health programme, who was acquainted with most of the international stakeholders due to prior interaction within previous capacity building initiatives.

Using a semi-structured interview guide with prompts, the interviewers invited course convenors to reflect on the structure and content of their courses, the mode of delivery and the barriers and facilitators to the recruitment and selection of students, delivery of course materials and assessment. Stakeholders were asked to provide information regarding the demand for a PGDip in their setting or network, public mental health training needs, desired impact of a PGDip programme and opinions regarding the structure and content of the programme.

### Data analysis

The interviews were audio-recorded and transcribed. The transcripts were imported into NVivo 12 software for analysis. Two researchers coded the data independently using thematic analysis [15], using both a deductive and an inductive approach to the data. The following steps were performed. The researchers familiarised themselves with the data and then coded a sample of the transcripts and compared codes. A coding template was developed, agreed upon and then applied to the remainder of the data, and adjusted appropriately throughout the process. The coding was compared using Cohen’s kappa to determine the inter-rater reliability in a randomly selected sample of transcripts (n = 10) from all three participant groups. Discrepancies were discussed and a kappa of 0.87 was achieved.

## Results

**Fig. 1:**
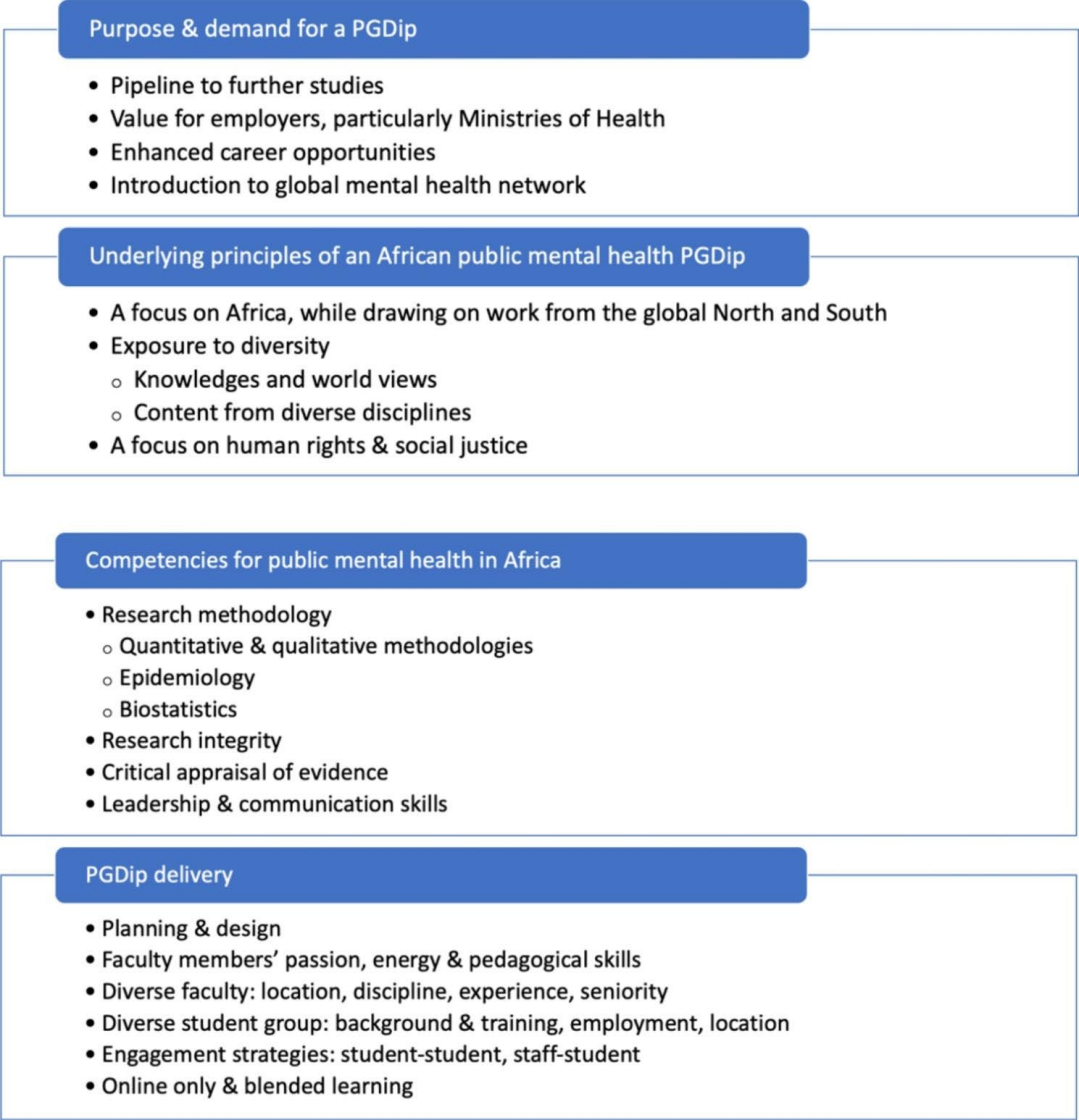
Coding tree

A number of themes and sub-themes emerged. See Fig. 1 below.

### Purpose & demand for a PGDip

Most participants expressed strong support for a PGDip, and viewed a PGDip programme, not only as a means to improve knowledge and skills for the workplace, but also as a “pipeline” or “stepping stone” to a Masters degree, and subsequent PhD degree. Despite the general support for a PGDip in Public Mental Health, one stakeholder mentioned the importance of integrating mental health into healthcare across settings and questioned whether a stand-alone mental health programme is necessary, arguing that mental health should rather be addressed more comprehensively within existing public health programmes.

A number of participants noted that the PGDip would be of value to employers, particularly ministries of health, and that such a programme would be well-placed to provide clinicians and managers with public mental health knowledge and fundamental research skills, to facilitate the translation of public mental health research and policy into practice within various healthcare settings, as one participant explained:

“There’s a real value in having people with research training working in ministries of health who know how to evaluate programs and implement evidence-based policy.” (Participant 30, stakeholder)

Others mentioned similar needs for advocates and implementers working in the civil society environment “because there is a lot of passion and … money but not enough skills to know how to plan their interventions or use the research evidence that is available to plan proper interventions that are sustainable.” (Participant 29, stakeholder)

Participants noted that, ideally, a PGDip qualification would lead to enhanced career opportunities for graduates, through the recognition of the value of the qualification from a leading African university. However, a few stakeholders felt that a PGDip qualification would not confer additional benefit for career prospects in their countries, where Masters and PhD programmes would be preferred. One participant explained:

“People would like to have higher incentive in doing this, and a diploma for a person who is developing his or her career in [country] may not be a really good incentive to give them additional pay or status rise.” (Participant 20, stakeholder)

Other stakeholders were of the opinion that being introduced to the global mental health network through a PGDip programme could benefit graduates’ careers, allow them to feel part of a broader community and “drive public mental health forward” by catalysing additional networks and collaborative partnerships. An additional benefit of a PGDip programme would be to capacitate “real time workers in the health system” whose “learnings get ploughed straight back into the health system”. By applying newly gained knowledge within their workplace, they would also build capacity of other people in their setting. Further, in healthcare settings, clinicians with public mental health expertise are vital in helping people to change and improve a system though collecting relevant data, identifying problems and contributing to a change process.

### Underlying principles of an African public mental health PGDip

Participants were invited to reflect on how the PGDip could play a role in addressing research knowledge gaps in public mental health in Africa, and a few key principles emerged. Respondents strongly emphasised that the PGDip be “Africa-focused” and build on existing knowledge from both the global north and the global south, while including areas known to be integral to wellbeing in Africa. Many noted the importance of encouraging students to grapple with different world views of mental health and the role that culture, religion and spirituality play in these conceptions. Reflecting on indigenous knowledge systems and mental health, one postgraduate course convenor advised the following:

“If one is able to invite some of these traditional healers to the classrooms or you do a fieldwork visit with the students … so that they can be in conversation with some of these knowledges. Not from a deficit point of view where we basically just listen to say ‘No, this is wrong, this is wrong,’ - you carry a red pen - but also how do we critically engage authentically and genuinely, and understand that all knowledges have limitations.” (Participant 9, SA course convenor)

In addition to highlighting the diversity of world views and knowledges, participants mentioned the need for diversity in content and views presented. Further, a common thread running through the course content should be inclusivity, regarding the contributions of all stakeholders in creating knowledge around mental health in Africa. Participants emphasised that the PGDip should encourage students to consider all stakeholders’ views and should foster a “deep respect for the perspective of the [mental health service] user”. Implicit in this approach is a value for human rights and a rights-based approach to mental health, and the importance of social justice, in particular its relationship to addressing social and structural determinants of health.

### Competencies for public mental health in Africa

Participants were asked to recommend content and focus areas for the PGDip curriculum. A strong theme emerged around building students’ research skills, and for the course to support students in developing a research protocol as a concrete output of the PGDip. A stakeholder emphasised that the research question should be guided by the student’s work experience and priorities in their immediate context:

“Many people have these wonderful ideas coming out of their practical work. And then they’re instructed that they must fill a gap in the literature … ‘This has never been done’ [should] not become the motivating reason for conducting a study. I think what is really important is ‘What problem does this solve?’ … ‘Why is this important knowledge to gain in this setting?’ Because people are doing the clinical work and don’t have the skills to do the research, and then gain the research skills but forget about all of these challenges that they saw in their clinical work and instead get immersed in gaps in the literature.” (Participant 30, stakeholder)

Suggestions for key competencies included knowledge of both quantitative and qualitative research methodologies, epidemiology and biostatistics, implementation science and research ethics, among others. Some participants encouraged the programme to focus on the appraisal of evidence and studies, while others emphasised the importance of operational research and basic skills in the monitoring and evaluation of programmes, as well as the skills needed to adapt interventions or develop culturally appropriate tools and interventions. One competency cited by a number of participants was the development of leadership skills, and attendant skills such as change management and communication skills. A course convenor described the core components of leadership:

“The focus would be on developing yourself as a leader and the reflective practice components around that. I would focus on that because it automatically forces people to think about how they relate to others around them, and so it deals with both managing the self and then managing and leading teams of people.” (Participant 25, SA course convenor)

### PGDip delivery

Course convenors shared a number of valuable insights from their experience which allowed the ARISE team to explore common facilitators to PGDip delivery and strategies to mitigate barriers experienced.

First, careful planning and design of course materials for online learning with the assistance of higher education design specialists are important to ensure implementation of “best practice for online learning”. Participants provided additional guidance on ensuring access for students with low bandwidth internet, warning that faculty should not be “be distracted by all the fancy things you can do” but should keep the site simple and provide course material that is easy to download for offline viewing. Further tips included limited use of video content and using online chat for discussions.

Second, programme staff’s “passion and energy” are vital to programme delivery, as well as lecturers’ pedagogical skills and openness to shifting to a teaching style suited to an online environment. Lecturers’ skills for creating content appropriate for online delivery were seen as important facilitators of effective learning. To this end, participants recommended choosing lecturers known to have good pedagogical skills, while also drawing in PhD students and junior academic staff to be trained in such skills. Further training in online teaching and facilitating was seen as crucial for programme success, particularly since most participants endorsed a ‘flipped classroom’ approach whereby students work through course material independently and attend facilitated group sessions to discuss material and apply their learning. Convenors recommended ongoing support for faculty members in the form of regular meetings to reflect on teaching practice and encourage peer review of teaching practices. Most convenors recommended an annual review of the programme and mentioned creative strategies to bring in topical information throughout the programme, such as using a discussion forum to draw students’ attention to new content and encourage engagement with the ideas.

Third, a diverse faculty exposes students to “more than one voice” involving many different perspectives and will enhance the PGDip in public mental health. Participants noted that online teaching provides opportunities to involve a wider group of lecturers. Participants recommended diversity in the teaching staff with regard to country, discipline, experience and seniority. Such diversity was seen as key in exposing the students to the multi-faceted field of public mental health and allowing students to understand the role of different stakeholders in improving mental health at a population level. Recommendations for teaching staff included experts from various fields, such as anthropology, social work, traditional healing and occupational therapy. Further, staff with a range of career and personal experience were suggested, including healthcare workers, community organisation staff, policymakers and people living with mental health conditions.

Fourth, recruiting a diverse group of students with regards to disciplinary background, job function and location not only encourages lively conversation and learning by “cross-pollination”, but also facilitates future interdisciplinary cooperation. A further benefit was described as broadening the scope of the public mental health field by encouraging the involvement of other professionals, in addition to the doctors, medical officers, nurses and psychologists who generally comprise the public mental health student body. Participants recommended that experienced health practitioners, civil society organisation staff and policymakers be included in the programme, with one participant saying that:

“Sometimes we forget the people on the ground, the people who are the champions you are trying to raise for mental health … In selection of candidates, [you should include] people who can actually make a difference within the system itself.” (Participant 29, stakeholder)

Fifth, engagement strategies are key for online learning to keep PGDip students engaged with each other, their lecturers and the course material. Course convenors highlighted lack of student engagement with lecturers and course material as a major barrier to online learning, with one participant saying that “you are lost, if you lose engagement with students”. Strategies included encouraging students to form groups on messaging platforms; lecturers using small group work to facilitate engagement between students and with the course material beyond the class sessions; and employing online chat rooms and discussion fora. Additionally, course convenors were emphatic that to effectively engage mature students, the course content and assessments should be relevant to students’ working environments. One of the commonly used strategies was employing ‘useful’ assessments, whereby students were tasked with developing policy briefs, quality improvement projects or research protocols. Such outputs could then be used in the workplace or to assist the student in moving through the postgraduate pipeline to Masters and PhD programmes.

Sixth, online learning can increase the accessibility of the programme for students from Africa and other LMICs. Some participants mentioned that the pandemic has shown that such approaches are possible and that students and lecturers have become more comfortable with the online learning environment. However, a number of course convenors advocated for a blended learning approach with initial face-to-face contact at the beginning of the programme in order to forms bonds and create a support network that endures throughout the programme. Barriers to such an approach included the constraints of the current COVID-19 pandemic and travel costs for students.

## Discussion

This research formed part of the planning process for the possible development of a PGDip in public mental health. Overall, we found that an Africa-focused PGDip programme is generally acceptable to stakeholders and could address the gap in public mental health research capacity in academic and operational contexts. The study also elicited a range of recommendations for the PGDip, including that: (i) the programme be guided by the overarching principles of human rights, social justice, diversity and inclusivity; (ii) the programme content reflect African public mental health needs within academic, policy and operational settings; (iii) PGDip faculty be appropriately skilled in teaching and developing material for online courses and (iv) the PGDip be carefully designed as a fully online or blended learning programme in collaboration with learning design experts, and include a focus on engagement strategies.

In deeming the PGDip plans acceptable, participants highlighted the limited resources and gaps in public mental health research capacity in Africa. Although investment in mental health research capacity building on the continent has increased in recent years, disparities in these areas remain evident [12, 16, 17], particularly in comparison with the substantial funding underpinning research and research training for infectious and other non-communicable diseases in the region [18, 19]. Further, most research capacity-building is undertaken within large research programmes [12, 20, 21], of which a small proportion are dedicated to mental health in African countries [22]. These initiatives are welcome additions to the isolated public mental health capacity building foci on the continent, including the CPMH postgraduate programmes. The addition of a foundational PGDip programme has the potential to broaden the reach of public mental health training and bring in the diverse voices needed in public mental health.

The recommendations made by participants regarding a rights-based, interdisciplinary, approach to mental health, inclusive of mental health service users and all other stakeholders, are aligned with the current thinking in global mental health, as outlined in the Lancet Commission on global mental health and sustainable development [23]. Further, there is a growing recognition in the field of the need to foreground culture and respond to local needs, including the social determinants of mental health [24–26]. The recent global shift to remote online learning has increased opportunity for inclusivity and diversity in faculty and students of higher education programmes, although barriers, such as technological challenges, still exist [27]. A postgraduate public mental health programme in Africa based on these principles will be well placed to influence academics, practitioners and policymakers in strengthening and designing inclusive, just, interdisciplinary approaches to mental health in their settings.

The key role of faculty in connecting students with content and designing active learning opportunities was highlighted by participants in this study and is a prominent theme in the higher education online teaching literature [28–30]. Faculty of online courses should be responsive and available, and strategies to enhance faculty presence, such as providing audio or video feedback on assignments, should be built into the course design [28, 29, 31]. Further, the lack of faculty training and support for developing the necessary competencies is a major barrier to the delivery of quality online programmes in higher education [32].

Additionally, participants strongly recommended careful, clear learning design and ongoing improvement, in collaboration with expert learning designers. The need for simple, easily navigable online content with clear instructions and expectations has been highlighted in the literature on online learning as important predictors of student satisfaction, engagement with learning and academic achievement [33, 34]. Certain important aspects of learning design include learning activities that are relevant to students’ practice in the workplace as well as the provision of synchronous and asynchronous opportunities for students to engage with course content, each other and faculty [33, 35, 36].

Despite the useful insights that emerged from this study, there are limitations which could limit the applicability of the findings. First, the participants were limited to stakeholders in public mental health and course convenors in public mental health and related disciplines. Broader engagement, including engagement with Ministries of Health in African countries, could provide additional insights into programme design and relevant content. Second, some learning barriers and associated solutions were not explored in detail and should be considered in further work. These include language barriers and challenging the current status quo of English as the ‘home language’ of public mental health [25], as well as infrastructure and resource issues in some institutions on the continent.

## Conclusions

The findings of this study have informed the curriculum design, as well as implementation and continuous quality improvement strategies to be employed in the launch of this new postgraduate public mental health programme. The findings have provided valuable insight into how to communicate key principles and skills suited to the rapidly developing public mental health field while keeping pace with changes in the higher education sphere.

## Data Availability

The data generated and analysed during the current study are not publicly available, due to the fact that these qualitative data inform the development of a specific academic programme and participants are easily identifiable due to their links with the programme. Additional quotes are available from the corresponding author on reasonable request. As a result, consent for data sharing was not included in the informed consent process.
